# Supplementary material for: Acteoside alleviates UUO-induced inflammation and fibrosis by regulating the HMGN1/TLR4/TREM1 signaling pathway
Source: PeerJ. 2023 Jan 18;11:e14765. doi: 10.7717/peerj.14765 (PMC9864189; doi:10.7717/peerj.14765)
Supplement: Supplemental Information 2 [file peerj-11-14765-s002.docx]

# Raw data

## Raw data of biochemical detection


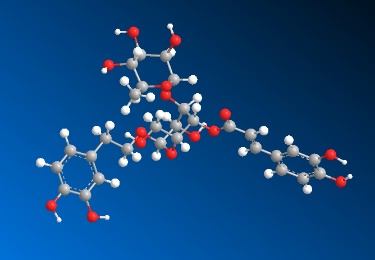












## Raw data of IHC

1. F4/80 (Sham/UUO+saline/UUO+Act):


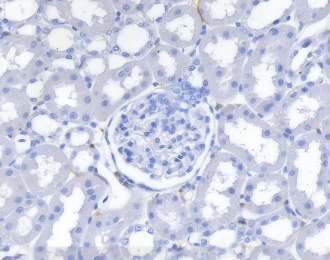

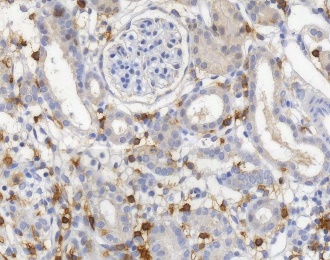

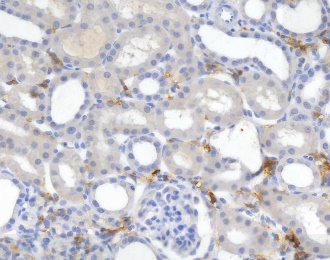


1. Mcp-1 (Sham/UUO+saline/UUO+Act):


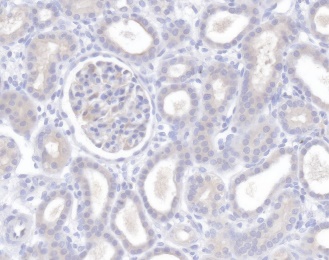

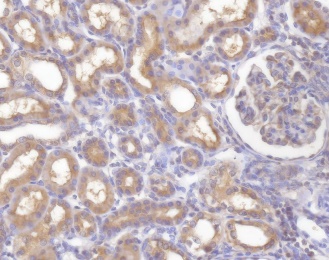

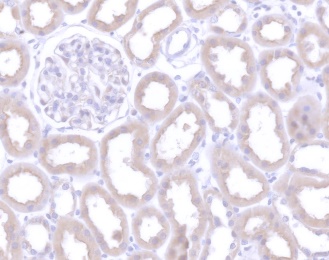


1. Kim-1 (Sham/UUO+saline/UUO+Act):


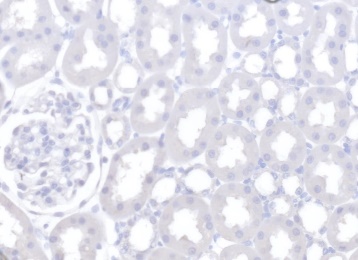

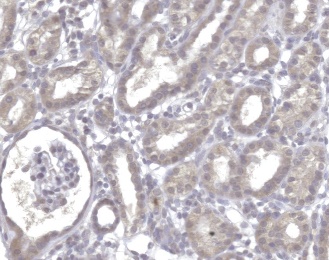

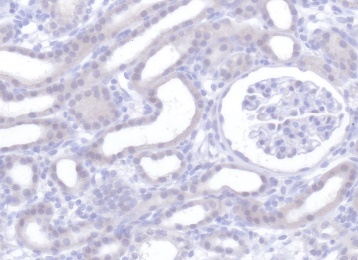


1. Alpha-SMA (Sham/UUO+saline/UUO+Act):


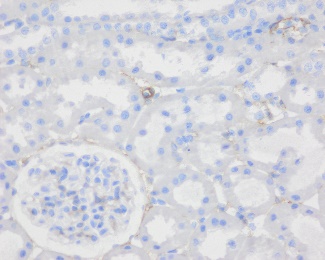

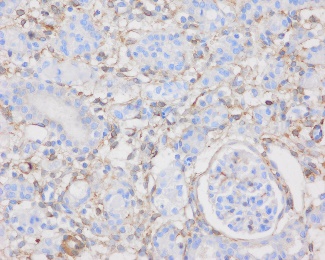

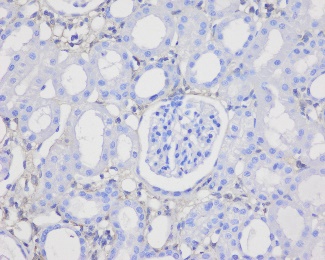


1. Bate-catenin (Sham/UUO+saline/UUO+Act):


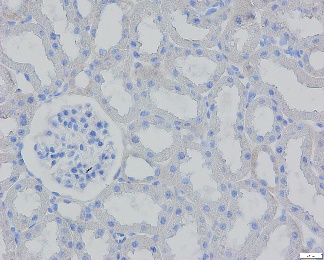

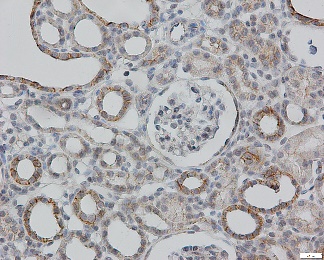

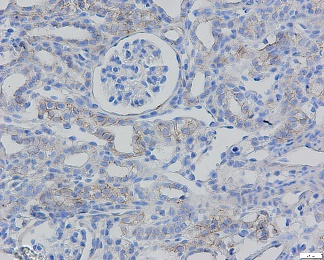


1. HMGN1 (Sham/UUO+saline/UUO+Act):


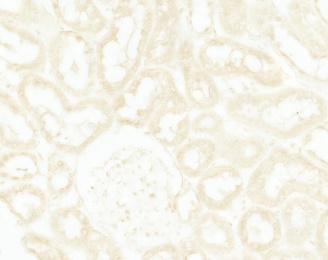

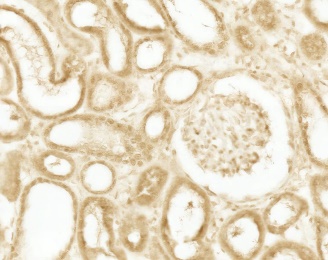

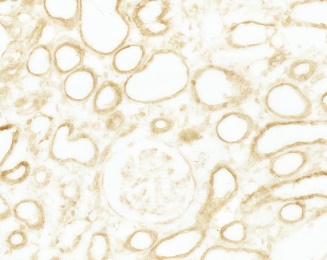


1. TLR4 (Sham/UUO+saline/UUO+Act):


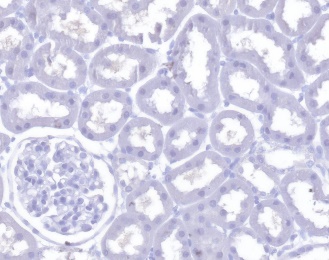

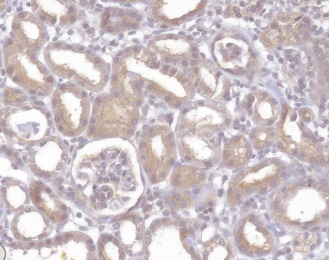

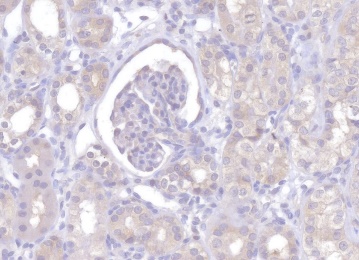


1. TREM-1 (Sham/UUO+saline/UUO+Act):


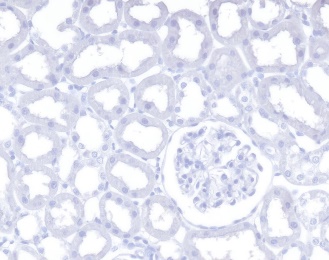

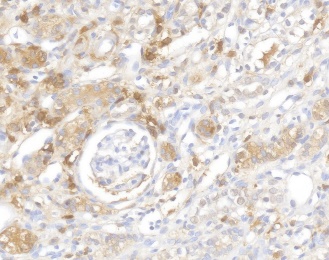

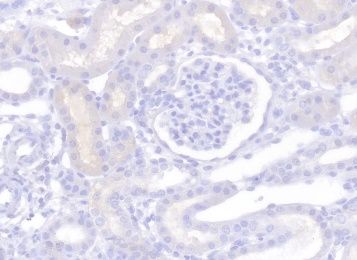


## Raw data of pathological staining

1. Raw data of HE (Sham/UUO+saline/UUO+Act):

( The magnification: 200×)


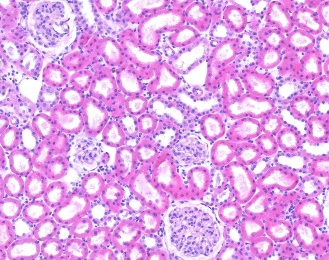

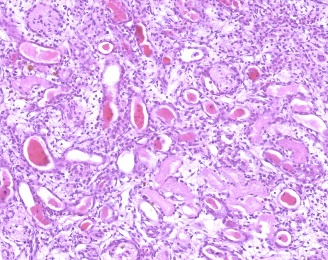

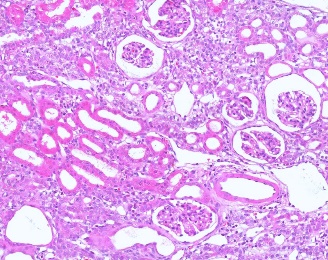


（The magnification: 400×）


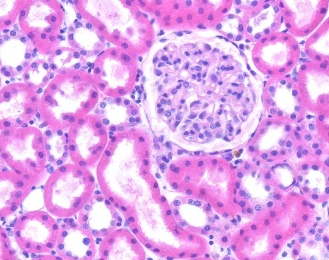

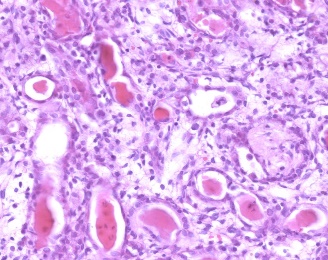

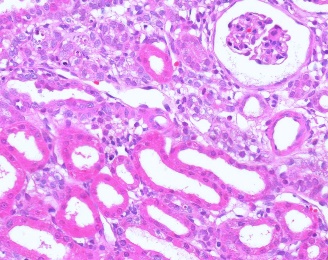


1. Raw data of Masson (Sham/UUO+saline/UUO+Act):

( The magnification: 200×)


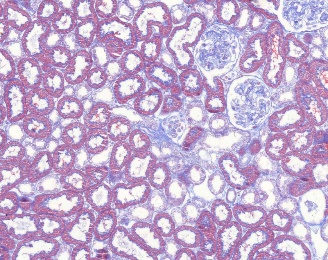

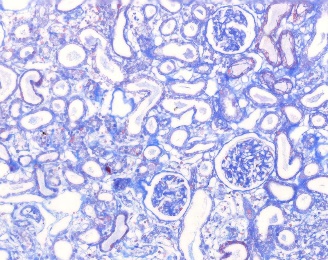

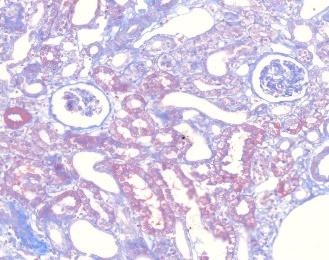


（The magnification: 400×）


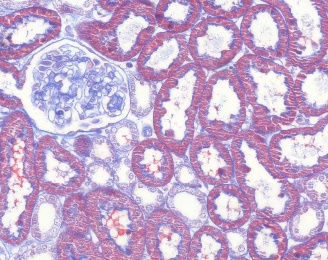

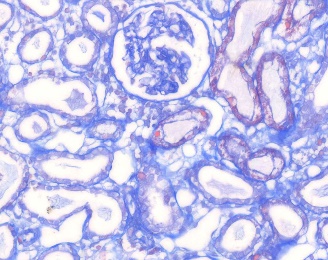

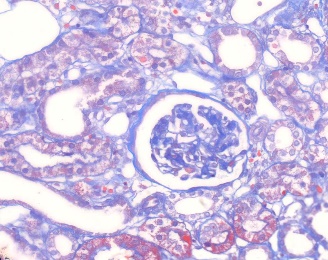


## Raw data of correlation analysis
